# A randomized, double-blinded, placebo-controlled, single dose analgesic study of preoperative intravenous ibuprofen for tonsillectomy in children

**DOI:** 10.3389/fped.2022.956660

**Published:** 2022-08-16

**Authors:** Xiaohuan Cui, Jianmin Zhang, Zhengzheng Gao, Lan Sun, Fuzhou Zhang

**Affiliations:** Department of Anesthesiology, National Center for Children's Health, Beijing Children's Hospital, Capital Medical University, Beijing, China

**Keywords:** postoperative pain, pediatric pain management, intravenous ibuprofen, tonsillectomy, non-steroidal anti-inflammatory drug

## Abstract

**Purpose:**

Tonsillectomy is a recognized treatment for children with tonsil hypertrophy and results in significant postoperative oropharyngeal pain. Fentanyl and other morphine-like analgesics are widely used as perioperative analgesia but are associated with side effects such as vomiting, nausea, and respiratory depression. As the least toxic non-steroidal anti-inflammatory drug, ibuprofen may be effective and safe for pain control after tonsillectomy. We aimed to explore whether the addition of intravenous (IV) ibuprofen administered at induction can reduce the need for early postoperativeanalgesics.

**Study design and methods:**

This randomized, double-blind, controlled clinical trial enrolled 95 pediatric patients who underwent tonsillectomy. Participants aged 6 months to 12 years were randomly assigned to either the experimental and control groups (1:1). The children were premedicated 15 min before surgery with IV ibuprofen 10 mg kg^−1^ or placebo (normal saline). Pain was scored at 15, 30, and 120 min after extubation, and IV fentanyl (0.5 mcg kg^−1^) was administered when the Faces, Legs, Activity, Cry, and Consolability (FLACC) Scale was ≥7 and deemed appropriate by the nursing staff in the post-anesthesia care unit (PACU). The visual analog scale was used as a supplementary evaluation for older children (≥7 years old) who were awake and could self-report pain. The primary outcome variable was the number of patients who received postoperative analgesia.

**Results:**

The requirement for rescue fentanyl was reduced by 18% with the addition of IV ibuprofen (*P* = 0.043). There were no signficant differences in the amount of fentanyl administered postoperatively (*P* = 0.127). Compared with the placebo group, the number of children who needed more than one dose of rescue fentanyl decreased in the experimental group, but the differences were not significant (*P* = 0.056). There were no significant differences between the groups in terms of operative blood loss (*P* = 0.978), vomiting, or postoperative bleeding (*P* = 0.474).

**Conclusion:**

It is safe to administer IV ibuprofen 15 min before tonsillectomy, and it can significantly reduce the need for rescue fentanyl. IV ibuprofen should be considered as an important part of the multimodal approach for postoperative analgesia in children.

**Clinical trial registration:**

Chictr.org.cn, identifier: ChiCTR2100044508.

## Introduction

Almost all children undergoing tonsillectomy experience considerable long-term pain and are afraid to swallow or eat because of severe throat pain. Adequate postoperative pain management in children remains challenging for several reasons, wherein the most essential is the lack of choices in pediatric analgesic drugs. Morphine-like analgesics such as fentanyl and sufentanil are widely used but are associated with a considerable incidence of nausea and vomiting. In addition, for children with obstructive sleep apnea syndrome, the above drugs may even increase the incidence of postoperative respiratory depression (1).

Non-steroidal anti-inflammatory drugs (NSAIDs) are an important component of multimodal pain management because of their anti-inflammatory effects (2). In addition, they are opioid-sparing and do not cause respiratory or central nervous system depression, making them an effective and safe option for children. However, there are some limiting factors in the use of NSAIDs for pediatric pain management. First, only a few NSAIDs are approved for perioperative analgesia in children, and “off-label” use is common in many clinical settings. Second, NSAIDs may interfere with platelet aggregation, thereby increasing the risk of bleeding. Third, most NSAIDs approved for children come in oral dosage forms and may increase preoperative gastric volume. Despite some concerns of postoperative bleeding, a previous study has shown that ibuprofen, a reversible NSAID, shows a good safety profile (3), and the American Academy of Otolaryngology-Head and Neck Surgery has specifically stated that “ibuprofen can be used safely for pain control after surgery” (4). Recently, long-awaited intravenous (IV) ibuprofen for pediatric analgesia has been approved by several countries, providing more options for postoperative analgesia management in children.

This randomized, double-blinded, placebo-controlled, single dose clinical trial evaluated whether IV ibuprofen used at the induction of pediatric tonsillectomy can reduce the use of postoperative analgesics and achieve an ideal analgesic effect.

## Materials and methods

The study strictly adhered to the Helsinki guidelines, and all the guardians signed informed consent before enrollment.

The inclusion criteria were: (1) age between 6 months and 12 years; (2) American Society of Anesthesiologists physical status classification I–III; and (3) scheduled for tonsillectomy with/without adenoidectomy.

The exclusion criteria were as follows: (1) use of analgesics such as NSAIDs, tramadol, or local anesthetics 24 h before study drug administration; (2) history of severe allergic illness or hypersensitive to any of the medications in the study; (3) significant cognitive impairment; (4) history of gastrointestinal diseases or active bleeding; (5) history of serious cardiovascular diseases; (6) dehydration or abnormal renal function; (7) active asthma; and (8) obstructive sleep apnea (obstructive apnea-hypopnea index >10 times per h). Children over seven years of age were familiarized with the Oucher visual analog pain (VAS) scale upon enrollment in the study.

After the assignment, participants were randomly assigned to two groups in a 1:1 ratio: the IV ibuprofen and placebo groups. Children in the first group were given a single dose of 10 mg kg^−1^ (maximum 400 mg) IV ibuprofen. In contrast, children in the other group were administered volume-matched normal saline at induction of anesthesia. The investigators, patients, surgeons, and nurses in the post-anesthesia care unit (PACU) were blinded to the intervention assignments.

Standard perioperative care was set in this study. After the children entered the operating room, monitoring including oxygen saturation, blood pressure, bispectral index, and electrocardiogram, would be established. Experimental fluid was administered before induction. General anesthesia was induced with 2 mcg kg^−1^ fentanyl, 2–3 mg kg^−1^ propofol, 0.1 mg kg^−1^ cisatracurium, and 0.5 mg kg^−1^ dexamethasone. The experimental fluid was prepared by a pharmacist and administered for 15 min. Lidocaine cream was applied to the tracheal catheters, and endotracheal intubation was performed after spontaneous ventilation had disappeared. Anesthesia was maintained with propofol 10 mg·kg^−1^·h^−1^ and remifentanil (0.3–0.4 mcg·kg^−1^·min^−1^) at the beginning and was adjusted to maintain the bispectral index monitor at 40–60. Systolic blood pressure change was maintained within 20% of the baseline values.

A fully trained team performed all surgeries. The procedure was initiated at least 15 min after the experimental fluid was administered, and tonsillectomy was performed using only low-temperature plasma ablation. At the end of the surgery, 0.1 mg kg^−1^ tropisetron was administered to all children to prevent postoperative nausea and vomiting. The operating time was defined as the time interval between placement and removal of the mouthpiece. The children were extubated when adequate spontaneous breathing was observed, and they were transferred to the PACU.

In the PACU, nurses would console the patients and evaluate how well they were waking up. At 15 and 30 min after surgery, all children were assessed using the Faces, Legs, Activity, Cry, and Consolability (FLACC) scale. For children over seven years of age who were awake enough to describe pain, the VAS scores were used as a supplement. Rescue fentanyl (0.5 mcg kg^−1^) was administered when the FLACC was ≥7, or the VAS was ≥70 mm. The nurses monitored the children intensively and reassessed them every 5 min. If the pain score persisted above 7, fentanyl injections were re-administered.

After returning to the ward, the children were discharged with cold compresses to the neck. They watched cartoons and drank normal-temperature water as soon as possible. Oral ibuprofen was allowed to be used as needed for at least 4 h after surgery. The incidence of vomiting, infusion site discomfort, and postoperative bleeding was followed up for 24 h.

The primary outcome variable was the number of children who received rescue fentanyl. Additional endpoints included the number of children who received repeat rescue fentanyl, weight-based postoperative fentanyl dose, intraoperative blood loss, pain scores at 15, 30, and 120 min after the surgery, the incidence of postoperative nausea and vomiting, and postoperative hemorrhage.

Data with a normal distribution are presented as the mean ± standard deviation, and the differences among groups were compared using the independent sample *t*-test (Student's *t*-test). Non-normally distributed data are expressed as the median (interquartile range), and the Mann–Whitney *U*-test was used to compare the differences. Categorical data were reported as numbers (percentages) and compared using the chi-square test or Fisher's exact test. Two-tailed *P*-values < 0.05 was considered statistically significant. Statistical analyses were performed using the SPSS statistical package (version 25.0; IBM SPSS Inc., Chicago, IL, USA).

A sample size of 80 participants was calculated using PASS 15.0 (NCSS PASS, UT, USA) to provide at least 80% power and a 0.05 significance level to detect a 30% reduction in the number of patients who needed supplementary fentanyl (5). Ninety patients were required to enroll, considering a 10% loss to follow-up rate.

## Results

**Figure 1:**
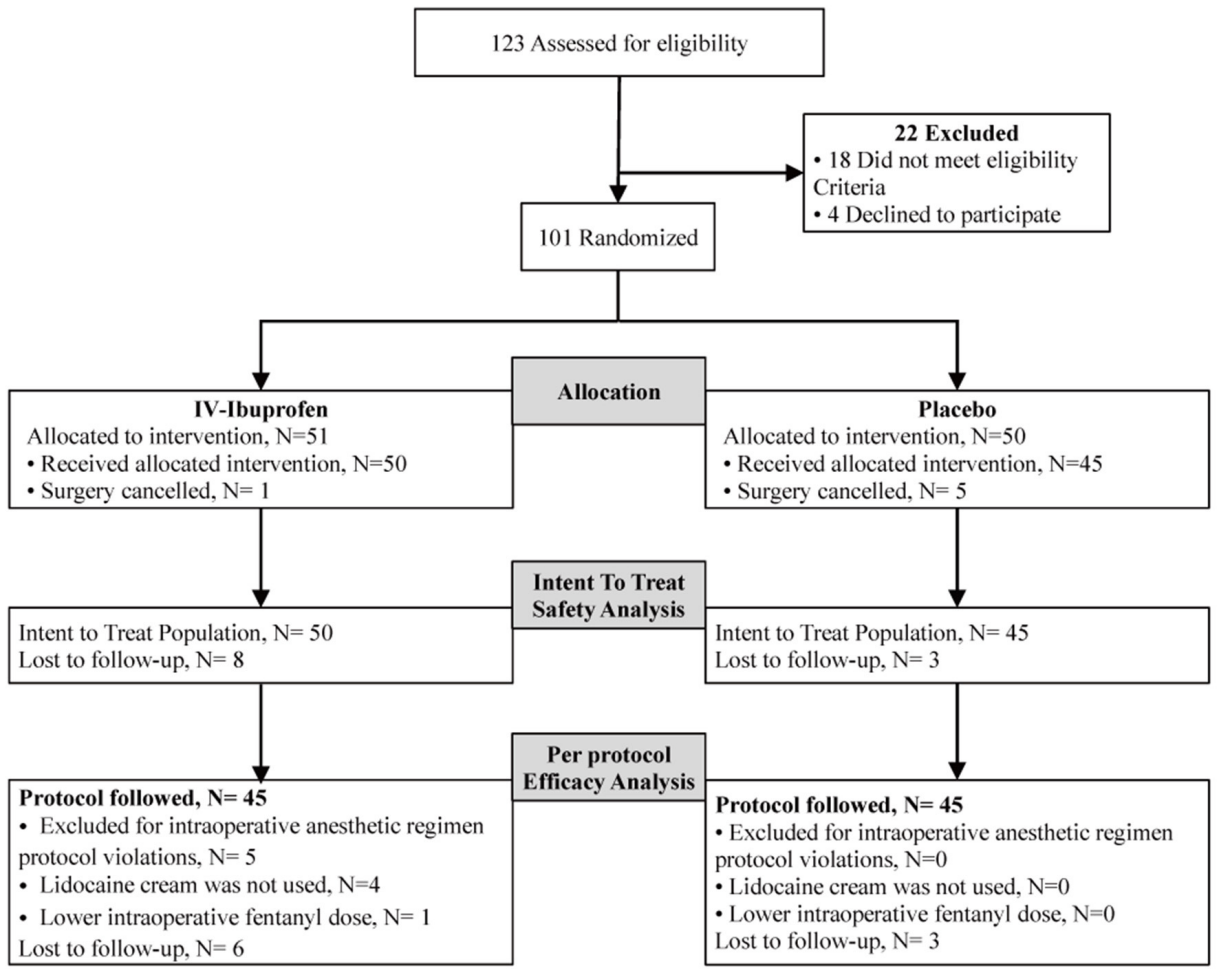
Participant flow.

A total of 123 children were screened, and 95 children were enrolled from March 19 to June 16, 2021. The flowchart diagram is illustrated in Figure 1. The distribution of the patient and surgical characteristics were not significantly different between the two groups (Table 1).

**Table 1:** Demographics.

	**IV-Ibuprofen**,	**Placebo**,
	***N* = 50**	***N* = 45**
**Gender**
Female	21 (42%)	19 (42%)
Male	29 (58%)	26 (58%)
*P*-value		0.456
**Age (years)**
Mean (SD)	6.1 (2.45)	6.5 (2.39)
Median	5.8	6.3
*P*-value		0.344
**Height (cm)**
Mean (SD)	116.9 (23.30)	122.0 (15.24)
Median	116.5	120.0
*P*-value		0.186
**Body weight (kg)**
Mean (SD)	26.0 (13.84)	26.9 (10.48)
Median	21.5	24.0
*P*-value		0.211
**BMI (kg/m2)**
Mean (SD)	17.1 (3.87)	17.4 (3.55)
Median	16.6	16.6
*P*-value		0.514
**Operation duration (min)**
Mean (SD)	33.0 (11.08)	33.3 (13.97)
Median	30.5	29.0
*P*-value		0.638
**Operation type**
tonsillectomy	7 (14%)	7 (16%)
adenotonsillectomy	43 (86%)	38 (84%)
*P*-value		0.437
**Types of tonsils**
Intracapsular	23 (46%)	20 (44%)
Extracapsular	27 (54%)	25 (56%)
*P*-value		0.446

**Figure 2:**
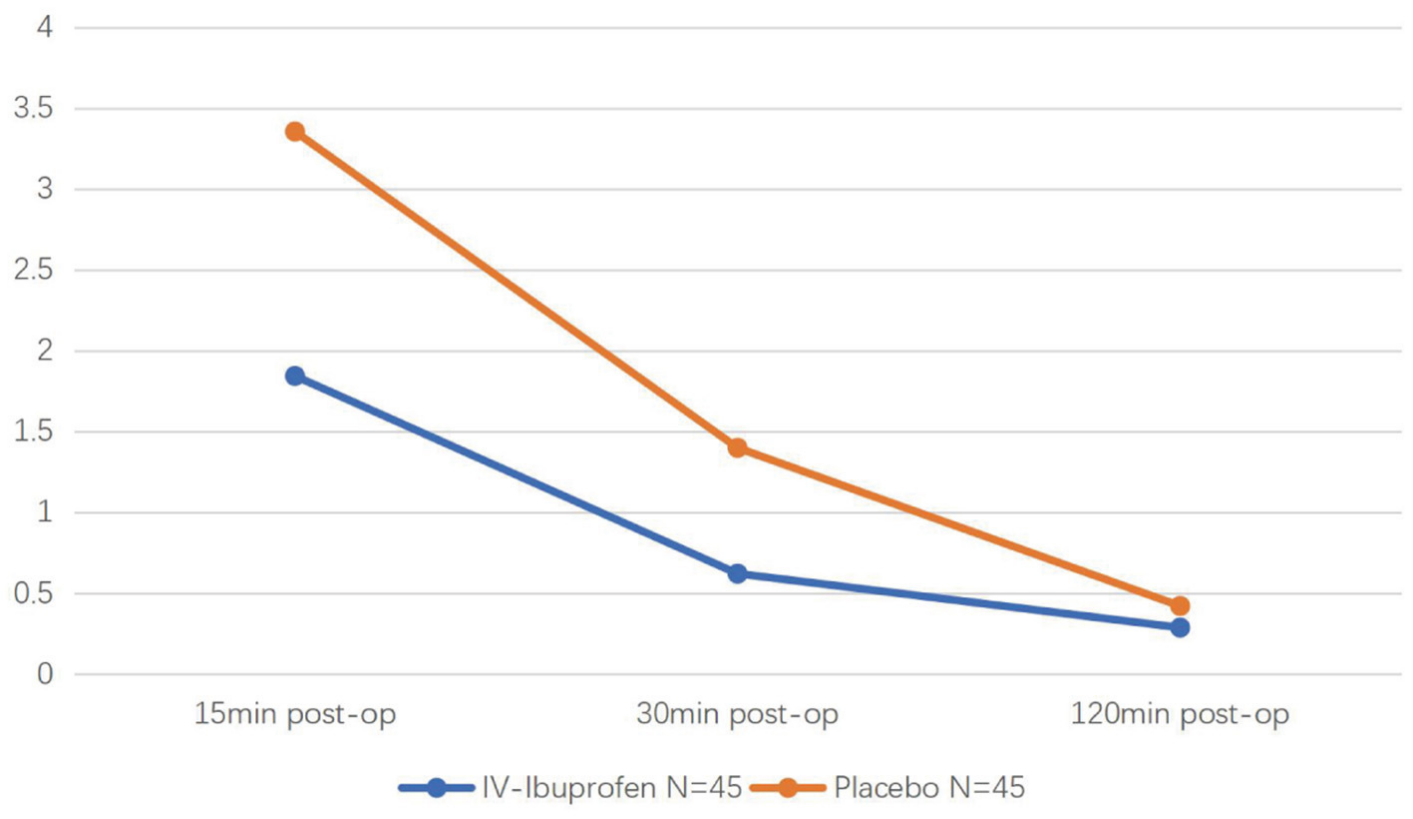
The Faces, Legs, Activity, Cry, and Consolability (FLACC) scale during the 2 h postoperative period of two groups.

Of the 95 children recruited, five were excluded because of protocol violations. Ninety children were eligible for efficacy (45 in the experimental group and 45 in the placebo group) (Figure 1). In the IV ibuprofen group, the number of children who received postoperative fentanyl was significantly lower than that in the placebo group [6 of 45 (13%) vs. 14 of 45 (31%), respectively, *P* = 0.043]. In addition, the pain scores of the two groups decreased with time after surgery, and significant differences were observed at 15 (*P* = 0.06) and 30 min (*P* = 0.08) (Figure 2). Although there were no significant differences in the amount of the supplementary fentanyl dose and the number of children receiving repeat rescue fentanyl, all five patients in the placebo group needed repeat fentanyl (Table 2).

**Table 2:** Postoperative analgesic requirements and intraoperative blood lose.

	**Intent to treat**		**Efficacy evaluable**	
	**Ibuprofen**	**Placebo**	**Ibuprofen**	**Placebo**
	**(*n* = 50)**	**(*n* = 45)**	**(*n* = 45)**	**(*n* = 45)**
**Number (%) of patients who received supplementary fentanyl**
0 dose supplementary fentanyl	40 (80%)	31 (69%)	39 (87%)	31 (69%)
≥1 dose supplementary fentanyl	10 (20%)	14 (31%)	6 (13%)	14 (31%)
*P*-value		0.139		0.043
**Number (%) of patients who received**
≤1 dose supplementary fentanyl	50 (100%)	40 (89%)	45 (100%)	40 (89%)
>1 dose supplementary fentanyl	0 (0%)	5 (11%)	0 (0%)	5 (11%)
*P*-value*(Fisher)		0.021		0.056
**Supplementary Fentanyl dose (μg kg** ^**−1**^ **)**
Mean (SD)	0.10 (0.20)	0.23 (0.39)	0.07 (0.17)	0.23 (0.39)
Median	0	0	0	0
*P*-value		0.128		0.127
**Surgical blood loss (ml)**
Mean (SD)	9.26 (2.11)	9.00 (2.67)	9.18 (2.21)	9.00 (2.67)
Median	10	10	10	10
*P*-value		0.781		0.978

Adverse events (AEs) are listed in Table 3. A total of 21 (22%) patients had AEs, wherein the most common AEs were infusion site discomfort (12%) and vomiting (4.2%). There were no differences between the two groups in surgical blood loss, perioperative bleeding, vomiting, infusion site discomfort, or reoperation rate.

**Table 3:** Adverse events.

	**Placebo**	**IV-Ibuprofen**	***P*-value**
	**(*N* = 45)**	**(*N* = 50)**	
	**No. of events**	**No. of events**	
Vomiting	3	1	0.536
Infusion site discomfort	5	6	0.892
Postoperative bleeding	1	0	0.474
Headache	0	0	—
stomachache	1	0	0.474
Rash erythematous	2	2	0.686
Hypoxia	0	0	—

^*^One patient in the placebo group experienced a bleeding-related adverse event but didn't need reoperation.

Tonsillar fossa hemorrhage was noted in one patient in the placebo group. He experienced nausea after leaving the PACU, spitting out ~5 ml of bloody saliva. An ear, nose, and throat specialist examined the patient and did not observe active bleeding. Therefore, he did not undergo resurgery, and the bleeding ended ~10 mins later.

## Discussion

Pain management after surgery in children remains challenging in many countries, mainly because of the following factors: first, the difficulty in pain assessment for children, especially in the early postoperative period; second, morphine-like analgesics, the essential component of pediatric postoperative pain treatment, may lead to several serious complications such as respiratory depression, sedation, and potentially apnea; and third, many NSAIDs are not authorized for pediatric use (6). Several studies have shown that pain management in children is usually inadequate (7–9). The precise use of drugs to achieve better analgesic effects is a common goal of pediatric anesthesiologists.

This was the first clinical trial evaluating the efficacy and safety of preoperative IV ibuprofen use in pediatric tonsillectomy in an Asian population. The analgesic activity of ibuprofen is related to its anti-inflammatory effects, and it can reduce the levels of cyclooxygenase (COX)-1 and COX-2-derived proteinoids in the blood (10). IV ibuprofen exerts its effects within 15 min, reaches the maximum concentration in 30 min (T_max_), and is metabolized by half in ~2 h. Therefore, for the pharmacology and pharmacokinetics analysis, we designed the study to determine whether IV ibuprofen used at least 15 min before surgery decreased the number of patients who received rescue fentanyl. The pain scores at 15 and 30 min were significantly lower in the IV ibuprofen group than in the placebo group. In addition, the number of children who needed rescue analgesia showed a significant reduction in the IV ibuprofen group. This indicates that IV ibuprofen has a preventive inhibitory effect on postoperative pain and is consistent with previous NSAID studies. Moreover, the number of children who required repeated rescue fentanyl and the weight-based amount of fentanyl in the two groups were similar. Notably, the cases requiring a second dose of rescue fentanyl were all from the placebo group [0 of 45 (0%) vs. 5 of 45 (11%), respectively, *P* = 0.056]; a larger sample size might lead to further results.

In children treated with preoperative IV ibuprofen, intraoperative or postoperative bleeding was not noted. Although there are concerns regarding the association between ibuprofen and perioperative hemorrhage, recent studies have shown that ibuprofen is safe for children undergoing tonsillectomy (11, 12). A meta-analysis involving 36 studies demonstrated no increased risk of bleeding in patients using NSAIDs after tonsillectomy (13). Similarly, a study including 6014 children found that when age is controlled, the incidence of post-tonsillectomy hemorrhage among patients treated with ibuprofen was not statistically increased compared to patients treated with codeine (14).

This trial's efficacy and safety findings are consistent with the postulate before the study and with those of several previous studies (1, 4, 6). This could provide more evidence for pediatric anesthesiologists to precisely manage postoperative pain in children and may reduce fentanyl dosage during induction.

Analgesia after tonsillectomy in children is a multi-pronged approach that includes pharmaceutical and non-pharmaceutical methods. During the first 15 min after awakening, it is difficult to differentiate between emergence delirium and pain in clinical practice (15). Therefore, nurses in the PACU console the children first when they cannot identify the cause of unsettling behavior. Other non-pharmaceutical measures, such as postoperative honey, ice lollipop, early drinking of water, and distraction, can also effectively relieve postoperative pain (2, 16). Rescue fentanyl was administered only to those who experienced severe pain (FLACC ≥7) to decrease the risk of opioid toxicity. The significant decrease in pain scores 2 h after surgery was associated with multimodal analgesic measures, including neck cold compression, early drinking, and watching cartoons. The pain scores were similar in the two groups 2 h after surgery, suggesting that ibuprofen was largely metabolized, which is consistent with the metabolic characteristics of ibuprofen.

An appropriate scale is essential for establishing baseline discomfort and measuring the response to treatment. In previous studies, different scales have been used to measure postoperative pain after tonsillectomy in children (1, 17–21). During the recovery period, children of all ages may not yet be able to express and quantify the degree of their pain; therefore, an observational tool seems more reliable. Voepel-Lewis et al. (22) conducted 73 observations on 29 ill adults and eight children to evaluate the reliability and validity of the FLACC. They found that “it can be used across populations of patients and settings, and the scores are comparable to those of the commonly used 0-to-10 number rating scale.” Pain assessment and management have become topics of interest (23), and further research is necessary to improve pain assessment in children after surgery.

The main limitation of this study was the anesthetic regimen containing fentanyl and remifentanil. This regimen has been used in our institution for years, and we retained it to reduce unnecessary exposure to postoperative pain. However, this may have clouded observations of the true effects of IV ibuprofen, which is more effective than the experimental results. Moss et al. (1) used less fentanyl in the induction period and maintained it with sevoflurane; significant reductions were observed in the doses of postoperative fentanyl, weight-based rescue fentanyl, and the number of patients who received more than one dose of fentanyl. Another limitation was the lack of age groups. In our study, infants younger than 6 months were excluded because previous research has shown that infants younger than 3–6 months metabolize analgesic medication differently than older children (24). The development of the nervous system in different stages of childhood may affect the way the body copes with pain and interventions. Previous studies suggested that postoperative pain is more severe in older children (25, 26). A large sample size with exact age stratification may have clarified the effects of IV ibuprofen more accurately and led to a more precise analgesic regimen for children. Third, this study only assessed the risk of postoperative bleeding in the first 24 h, which means that late bleeding events may have been missed. We intended to design a further study with an extended follow-up time of 14 days to observe possible bleeding caused by ibuprofen.

In conclusion, IV ibuprofen has a preventive inhibitory effect on pediatric postoperative pain and does not increase perioperative bleeding.

## Data availability statement

The raw data supporting the conclusions of this article will be made available by the authors, without undue reservation.

## Ethics statement

The studies involving human participants were reviewed and approved by the Clinical Research Ethics Committee of Beijing Children's Hospital. Written informed consent to participate in this study was provided by the participants' legal guardian/next of kin.

## Author contributions

XC and ZG designed the study. XC analyzed the data and wrote the paper. LS and FZ participated in the interpretation of data. JZ directed the research and offered some suggestions. All authors contributed to the article and approved the submitted version.

## Conflict of interest

The authors declare that the research was conducted in the absence of any commercial or financial relationships that could be construed as a potential conflict of interest.

## Publisher's note

All claims expressed in this article are solely those of the authors and do not necessarily represent those of their affiliated organizations, or those of the publisher, the editors and the reviewers. Any product that may be evaluated in this article, or claim that may be made by its manufacturer, is not guaranteed or endorsed by the publisher.

## Abbreviations

AE: Adverse events
COX: cyclooxygenase
FLACC, Faces, Legs, Activity, Cry: and Consolability
IV: intravenous
NSAID: non-steroidal anti-inflammatory drug
PACU: post-anesthesia care unit.
